# Optimization of *Bacillus cereus* Fermentation Process for Selenium Enrichment as Organic Selenium Source

**DOI:** 10.3389/fnut.2020.543873

**Published:** 2020-11-05

**Authors:** Xujun Chen, Shuyi Li, Xin Cong, Tian Yu, Zhenzhou Zhu, Francisco J. Barba, Krystian Marszalek, Czesław Puchalski, Shuiyuan Cheng

**Affiliations:** ^1^National R&D Center for Se-rich Agricultural Products Processing, College of Food Science and Engineering, Wuhan Polytechnic University, Wuhan, China; ^2^Enshi Se-Run Health Tech Development Co., Ltd, Enshi City, China; ^3^Nutrition and Food Science Area, Department of Preventive Medicine and Public Health, Food Science, Toxicology and Forensic Medicine, Faculty of Pharmacy, Universitat de València, Avda. Vicent Andrés Estellés, València, Spain; ^4^Department of Fruit and Vegetable Product Technology, Prof. Wacław Dabrowski Institute of Agricultural and Food Biotechnology, Warsaw, Poland; ^5^Department of Chemistry and Food Toxicology, Institute of Food Technology and Nutrition, College of Natural Sciences, University of Rzeszów, Rzeszow, Poland; ^6^Department of Bioenergetics, Food Analysis and Microbiology, Institute of Food Technology and Nutrition, College of Natural Sciences, University of Rzeszów, Rzeszow, Poland

**Keywords:** selenium enrichment, *Bacillus cereus*, fermentation, response surface methodology, organic selenium

## Abstract

Selenium is an essential trace element and micronutrient for human health. Application of organic selenium in plants and microorganisms as trace element supplement is attracting more and more attention. In this study, *Bacillus cereus*, an important probiotic, was used for selenium enrichment with sodium selenite as selenium source. The growth curve of *B. cereus* was investigated, and 150 μg/ml was selected as the concentration of selenium for *B. cereus* fermentation. With application of response surface methodology, the optimal fermentation conditions were obtained as follows: inoculation quantity of 7%, culture temperature of 33°C, and shaking speed of 170 rpm, leading to the maximal selenium conversion ratio of 94.3 ± 0.2%. Field emission scanning electron microscope and energy dispersive spectrometry evidenced that inorganic selenium had been successfully transformed. This study may contribute to get a strain with high Se conversion ratio, so as to extract organic selenium in the form of selenoprotein to be used for further application.

## Introduction

As an essential trace element, as well as a kind of micronutrient, selenium (Se) was first discovered in 1817 by Swedish scientists in the sludge of leaden chambers used for production of sulfuric acid. Selenium plays a key role in maintaining the balance of human immune, endocrine, metabolism, and intracellular environment (1). Especially in the immune system, selenium can bind to erythrocyte, albumin, and plasma globulin and be transported to tissues after being absorbed by organisms, and 28 to 46% of selenium can deposit in skeletal muscle (2). Selenium is also an important component of the glutathione peroxidase (GSH-px) subunit, which can detoxify free radicals and protect the body from injury (3). Deficiency of selenium in humans can lead to a series of adverse health conditions, such as Keshan disease, Kashin-Beck disease, poorer immune function, and problematic fertility/reproduction (4). Selenium can be roughly divided into inorganic selenium and organic selenium. Inorganic selenium is mainly composed of selenate and selenite with the characteristics of low bioavailability and toxicity when in excess (5), whereas organic selenium contains a variety of selenium amino acids (such as selenomethionine and selenocysteine) (6), and it can provide antioxidant benefits by acting both as direct antioxidants and as a source of selenium for synthesis of selenium-dependent antioxidant and repair of proteins (e.g., glutathione peroxidases, thioredoxin reductases, and methionine sulfoxide reductases) (7). In recent years, researches on biological transformation of selenium have begun to attract much attention. Two methods of bioconversion of selenium have been reported: microbial biotransformation and selenium-enriched sprouted seeds via sprouting (8). Fermentation of cultures of yeasts and lactic acid bacteria for selenium enrichment have been studied a lot recently, showing that bioconversion by microorganisms was faster and safer than selenium-enriched sprouted seeds.

Previous studies have shown that some bacteria can convert inorganic selenium into organic selenium in the form of protein (9). Hong et al. (10) studied the effects of different selenium concentrations on the growth of *Fusarium* spp., and results showed that adding selenium within the concentration of 0.10–1.00 mg/L could evidently promote the mycelial growth of *Fusarium tricinctum*. Yuan et al. (11) optimized the medium formulation of beer yeast by single factor and orthogonal test and prepared selenium-enriched beer yeast with a total selenium content of 23 mg/L. Low selenium concentration can affect the growth of *Streptococcus acidophilus* bacteria, the bioaccumulation of selenium and the form of selenium. By increasing the concentration of sodium selenite, the dry weight of *S. acidophilus* can be significantly increased. At the same time, selenium can bind to bacterial proteins and form selenocysteine (12). *Bacillus cereus*, an endospore-forming, gram-positive bacterium, is of great economic importance due to its ability to produce various enzymes, such as amylase (13), protease (14), and cellulase (15). As probiotics, *B. cereus* can produce many beneficial effects for organisms. Using *B. cereus* as a feed additive can affect the intestinal immune system of piglets after feeding and enhance the double-positive cell population of cd8/cd3 in the intestinal epithelium of a probiotics group (16). However, to our best knowledge, selenium enrichment in *B. cereus* via fermentation has been barely studied.

The purpose of this study was to investigate the selenium enrichment ability of *B. cereus*. The effect of selenium concentration in the form of sodium selenite on selenium transforming during *B. cereus* fermentation was studied. The fermentation parameters such as inoculation quantity, culture temperature, and shaking speed were optimized to obtain the optimal selenium conversion ratio. The surface characterization of selenium-enriched *B. cereus* was carried out using field emission scanning electron microscope and energy dispersive spectrometry.

## Materials and Methods

### Strain Material

The experimental *B. cereus* strains were isolated from soil that was taken from Enshi (China) and obtained by Se-Run Health Tech Development Co., Ltd., in Enshi (China). The phylogenetic tree of the strain obtained from 16S rDNA sequencing analysis showed that the homology of the strain with *B. cereus* strain reached 99%. The strain was collected at −80°C and inoculated into Luria-Bertani (LB) agar medium plates and activated at 37°C for 6 h, and then single colonies were selected from it and inoculated into liquid LB medium. The strain seed liquid was obtained by culture at 150 rpm on a shaking incubator HZ-150L (Ruihua Instrument & Equipment, China) with a rotational radius of 20 mm (same rotational radius below) for 12 h, and the concentration is 3 × 10^7^ cfu/ml.

### Chemicals and Reagents

The bacterial genomic DNA extraction kit was purchased from Biovision (Milpitas, California, USA). The Gel Extraction Kit was purchased from Qiagen (Germany). The DNA agarose gel Recovery Kit was purchased from Solarbio (Beijing, China). Analytical grade sodium selenite, analytical grade hydrochloric acid sodium chloride, and ethylenediaminetetraacetic acid disodium salt (EDTA-2Na) were purchased from Sinopharm Chemical Reagent Co., Ltd. (Shanghai, China). O-phenylenediamine was purchased from Aladdin Biochemical Technology Co., Ltd. (Shanghai, China). Tryptone was purchased from OXOID (Hants, UK). Yeast extract was purchased from Aoboxing Biotechnology (Beijing, China). Agar was purchased from Biofroxx (Germany).

### Determination of Strain Growth Curve

Strain seed liquid was inoculated into 50 ml liquid LB medium with 5% inoculation amount and cultivated at 37°C and 150 rpm. The absorbance of *B. cereus* at 600 nm was measured per 1 h by the growth curve tester Bioscreen C (Finland) to obtain the growth curve.

### Optimization of *B. cereus* Fermentation for Selenium Enrichment

#### Optimization of Fermentation Conditions for Selenium Enrichment

Sodium selenite (Na_2_SeO_3_), source of selenium, was dissolved in distilled water (10 mg/L) and sterilized by high-pressure steam. Strain seed liquid was inoculated into LB liquid medium with 5% inoculation volume (total volume 50 ml), and sodium selenite was added during logarithmic growth period (4 h) with the concentration of 150 μg/ml, according to previous tests to assure sufficient source selenium content. The fermentation duration was 24 h. On the basis of the single factor tests, Box-behnken experimental design was used to optimize the fermentation conditions. The strain seed liquid was inoculated into LB liquid medium with an inoculation amount of 1~9%, and then culture temperature was set at 29~37°C with shaking speed of 110~190 rpm. The coding and actual level of independent variables of the process are shown in Table 1. The experimental runs performed are listed in Table 2.

**Table 1:** The code levels of investigated parameters.

**Variables**	**Code level**		
	**−1**	**0**	**1**
Inoculation amount (*X*_1_), %	5	7	9
Culture temperature (*X*_2_), °C	31	33	35
Shaking speed (*X*_3_), rpm	150	170	190

**Table 2:** Response surface analysis test design and results of selenium conversion ratio.

**Run**	**Coded variables**			**Actual variables**			**Selenium conversion ratio (%)**
	***A***	***B***	***C***	***X*_**1**_**	***X*_**2**_**	***X*_**3**_**	***Y***
1	1	1	0	9	35	170	0.705
2	0	1	1	7	35	190	0.720
3	1	0	−1	9	33	150	0.797
4	1	−1	0	9	31	170	0.651
5	−1	0	−1	5	33	150	0.814
6	−1	−1	0	5	31	170	0.702
7	0	1	−1	7	35	150	0.717
8	0	0	0	7	33	170	0.962
9	0	−1	1	7	31	190	0.640
10	0	0	0	7	33	170	0.927
11	0	0	0	7	33	170	0.905
12	0	0	0	7	33	170	0.908
13	−1	0	1	5	33	190	0.769
14	−1	1	0	5	35	170	0.732
15	0	0	0	7	33	170	0.906
16	0	−1	−1	7	31	150	0.701
17	1	0	1	9	33	190	0.722

*A and X_1_ represent inoculation amount (%); B and X_2_ are the culture temperature (°C), and C and X_3_ are the shaking speed (rpm)*.

The selenium conversion ratio (*Y*) was fitted to a quadratic regression model for response surface analysis, as shown in Equation (1):

(1) $$\begin{array}{l} Y=\beta_{\text{0}}\text{+}\beta_{1}A+\beta_{2}B+\beta_{3}C+\beta_{4}AB+\beta_{5}AC+\beta_{6}BC+\beta_{7}A^{2}\\ \;+\beta_{8}B^{2}+\beta_{9}C^{2} \end{array}$$

Where *A, B*, and *C* correspond to the coded independent variables, namely, inoculation amount, culture temperature, and shaking speed. The β_0_ ~ β_9_ values represent the corresponding regression coefficients. The experiments were randomized to maximize the effect of unexplained variability on observed responses due to exogenous factors.

#### Determination of Selenium Conversion Ratio

The residual inorganic selenium content in the fermentation solution was analyzed according to Zeng et al. (17). Briefly, the cultured strain liquid was centrifuged for 20 min at 2,415 g, then the absorbance value of the supernatant was measured at 600 nm (OD_600nm_) to determine the inorganic selenium content by applying the standard curve equation. The selenium conversion ratio (*R*_Se_) was calculated as follows:

(2) $$\begin{array}{l} R_{Se}=\frac{Se_{t}-Se_{i}}{Se_{t}}\times 100\% \end{array}$$

where *Se*_*t*_ is the total selenium content (mg) in 50 ml medium, and *Se*_*i*_ is the inorganic selenium content (mg) in 50 ml medium after fermentation.

### Surface Element Analysis of Selenium-Enriched *B. cereus*

The strain liquid after fermentation was centrifuged at 2,415 g for 20 min, then the supernatant was discarded and the precipitate was washed with ~35 ml sterile water to remove the residue selenium in the solution or adsorbed at the surface of strain. Then the freeze-dried strain powder (with or without selenium enrichment) was pasted on the copper sheet with conductive adhesive and sprayed with gold. Then the surface morphology was analyzed by field emission scanning electron microscope (SEM) (SIGMA 500, Carl Zeiss AG, Germany) under accelerated voltage of 3 kV, and the surface elements in the field of vision were qualitatively observed by energy dispersive spectrometry (EDS).

### Statistical Analysis

All trials were carried out in triplicate, and all the data were reported as means ± SD (standard deviation). The statistical significance was evaluated using Student's *t* test, and *p* < 0.05 or 0.01 was taken as significant.

## Results and Discussion

### Determination of Growth Curve

**Figure 1:**
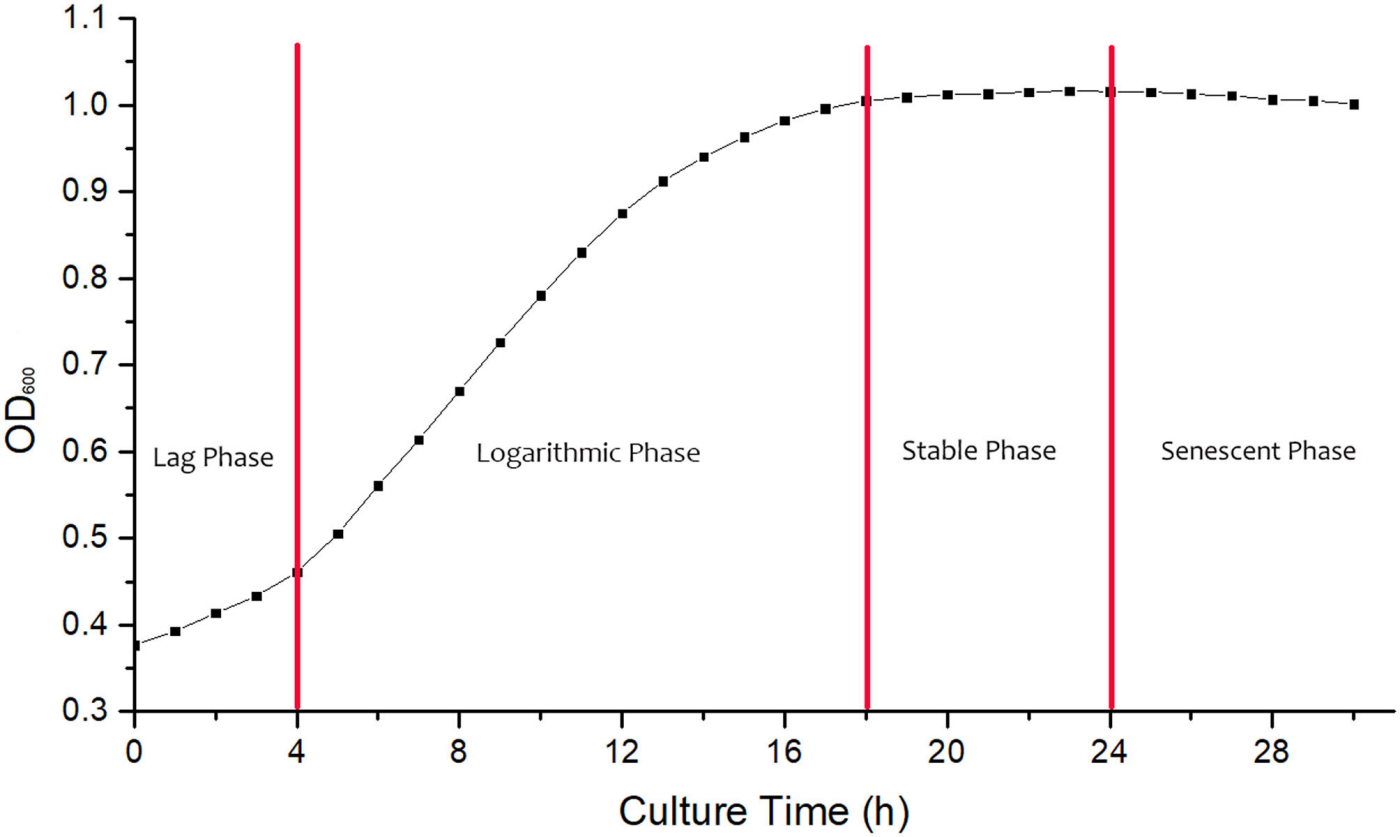
The growth curve of strain in culture.

The growth curve of *B. cereus* is shown in Figure 1. The strain was adapting to the new culture environment in lag phase at 0–4 h, when the increase of absorbance value was not obvious. At 4 h, the bacteria begin to enter a logarithmic growth phase, when the bacteria metabolize vigorously and the transformation rate is high; the absorbance value begins to increase significantly. At 24 h, the absorbance value of bacterial solutions began to decrease, and then the bacteria entered the senescent phases, with a large number of autolysis and low transformation rate. Suhajda et al. found that adding inorganic selenium in prophase of the logarithmic phase resulted in the highest selenium content of yeast cells. Moreover, with the proliferation of cells in the logarithmic phase, Se enrichment ability increased and then gradually weakened as the stable phase approached (18). Jin et al. (19) also found that adding sodium selenite at the initial stage of the logarithmic phase could obtain the maximum selenium content and selenium conversion rate of strain. Congcong et al. studied the effect of different selenium addition time on the total selenium content of yeast and *Lactobacillus*, and the results showed that adding sodium selenite in prophase of the logarithmic phase could obtain a higher total selenium content than in the prophase of growth stage and the metaphase and anaphase of logarithmic phase (20). Hong et al. (21) found that the organic selenium content and selenium conversion ratio of *Enterobacter mori* first increased and then decreased with selenium addition time and reached the maximum in the logarithmic growth phase, then decreased in the senescent phases. Therefore, the 4 h logarithmic growth period was chosen as the time of adding sodium selenite, and the culture time was 24 h.

### Optimization of Fermentation Process for Selenium Enrichment

It can be seen from Table 3 that the overall model *p* value is < 0.0001, demonstrating that the regression equation was very significant. The lack of fit value (*p* value = 0.8266 > 0.05) suggested that it was an adequate model to accurately predict the response variable. The coefficient *R*^2^ = 0.9836 also indicated the goodness of the model for selenium conversion ratio. Regression analysis showed that *A*^2^, *B*^2^, and *C*^2^ have a very significant effect on the selenium conversion ratio (*p* < 0.01), and *A, B*, and *C* have a significant effect on the protein extraction rate (*p* < 0.05). Among the three variables, culture temperature was the most significant factor affecting the selenium conversion ratio, according to the *p* value. The next most significant factor was the shaking speed, followed by the inoculation amount.

**Table 3:** ANOVA analysis of experimental data.

**Source**	**Sum of squares**	**Df**	**Mean square**	***F* value**	***p* value Prob > *F***	**Significance**
Model	0.17	9	0.019	46.69	<0.0001	^**^
A	0.00253	1	0.00253	6.31	0.0403	^*^
B	0.00404	1	0.00404	10.07	0.0156	^*^
C	0.00397	1	0.00397	9.90	0.0162	^*^
AB	0.000162	1	0.000162	0.40	0.5452	N
AC	0.000224	1	0.000224	0.56	0.4789	N
BC	0.000990	1	0.000990	2.47	0.1603	N
*A*^2^	0.022	1	0.022	53.75	0.0002	^**^
*B*^2^	0.098	1	0.098	243.93	<0.0001	^**^
*C*^2^	0.023	1	0.023	58.35	0.0001	^**^
Residual	0.00281	7	0.000401			
Lack of fit	0.000512	3	0.000171	0.30	0.8266	N
Pure error	0.00230	4	0.000574			
Cor total	0.17	16				
*R*^2^	0.9836					
Adjusted *R*^2^	0.9625					

*A represents inoculation amount (%); B represents culture temperature (°C), and C represents shaking speed (rpm). In the above table*,

** means very significant difference when p < 0.01;

* *represents the significant difference when p < 0.05, and N is not significant at p > 0.05*.

**Figure 2:**
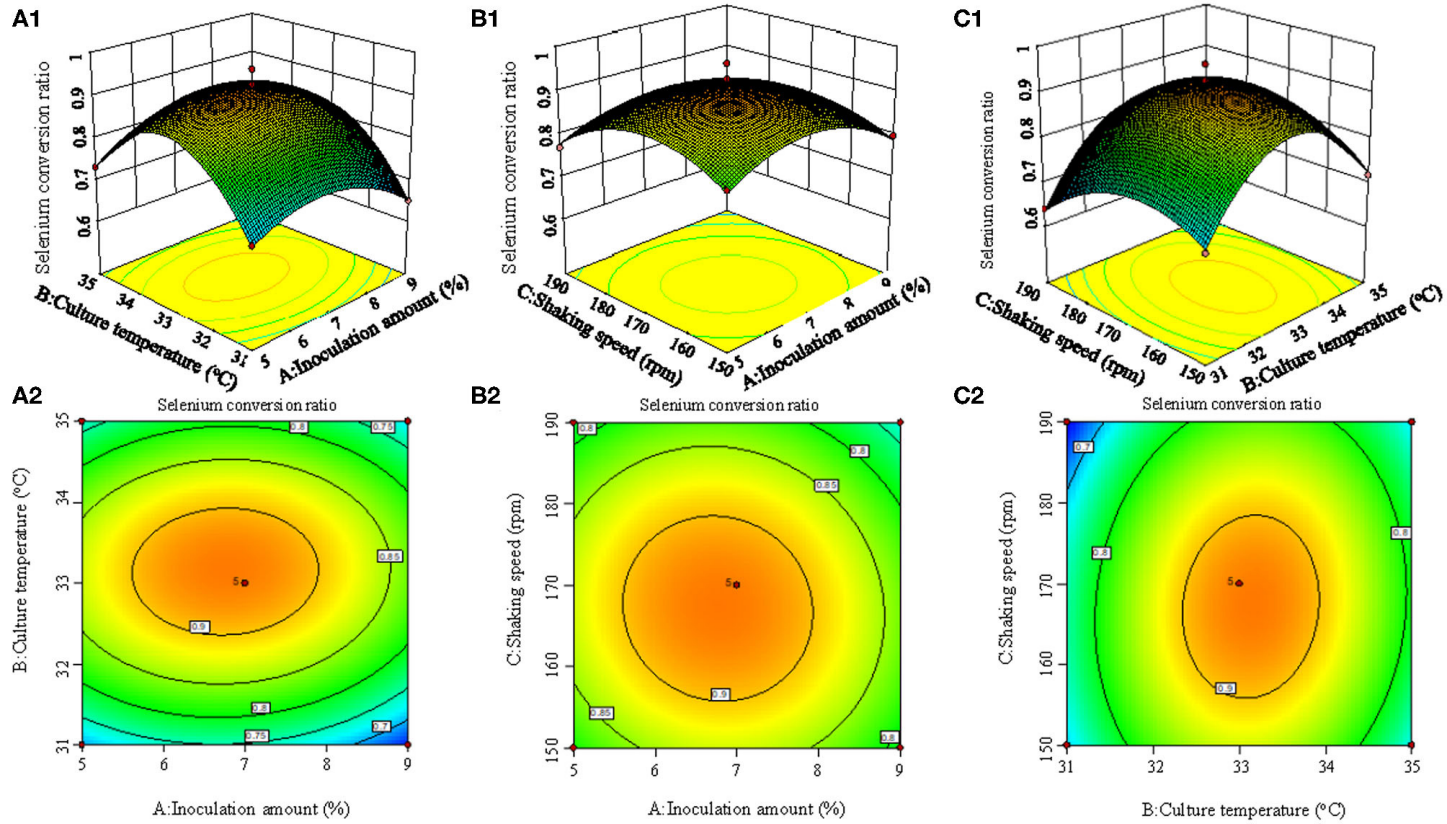
3D response surface plots **(A1, B1, C1)** and contour plots **(A2, B2, C2)** showing the interaction effects of inoculation amount and culture temperature **(A1, A2)**, inoculation amount and shaking speed **(B1, B2)**, and culture temperature and shaking speed **(C1, C2)** on the selenium conversion ratio.

Figure 2 shows the 3D surface response plots, representing the complex interaction between fermentation conditions on selenium conversion ratio. When the inoculation amount increased from 1 to 7%, the selenium conversion ratio was augmented from 58 to 83.6%. However, further increase of inoculation amount led to lower selenium conversion ratio. The increase of culture temperature and shaking speed led to the same trend regarding the variation of selenium conversion ratio. This result could be attributed to the fact that when inoculation amount, culture temperature, and shaking speed was beyond a certain range, the growth of strain may be inhibited.

According to the experimental data and model analysis, the maximum selenium conversion ratio of the strain (92.5%) could be achieved under the optimal conditions: inoculation amount 6.772%, culture temperature 33.130°C, and shaking speed 167.263 rpm. In order to verify the availability and reliability of the regression model obtained in the response surface test design, the above optimal preparation parameters were used to verify the test. To facilitate the process of the experimental operation, the inoculation amount used was 7%, the culture temperature was 33°C, and the shaking speed was 170 rpm, and the three measurements were performed in parallel. In the verification experiments, the selenium conversion ratio was 94.3 ± 0.2%, which was close to the predicted value, indicating that the predicted model agrees well with the actual situation. However, the form of organic selenium in the bacteria still needs further characterization.

### Surface Element Analysis of Selenium-Enriched Strains

**Figure 3:**
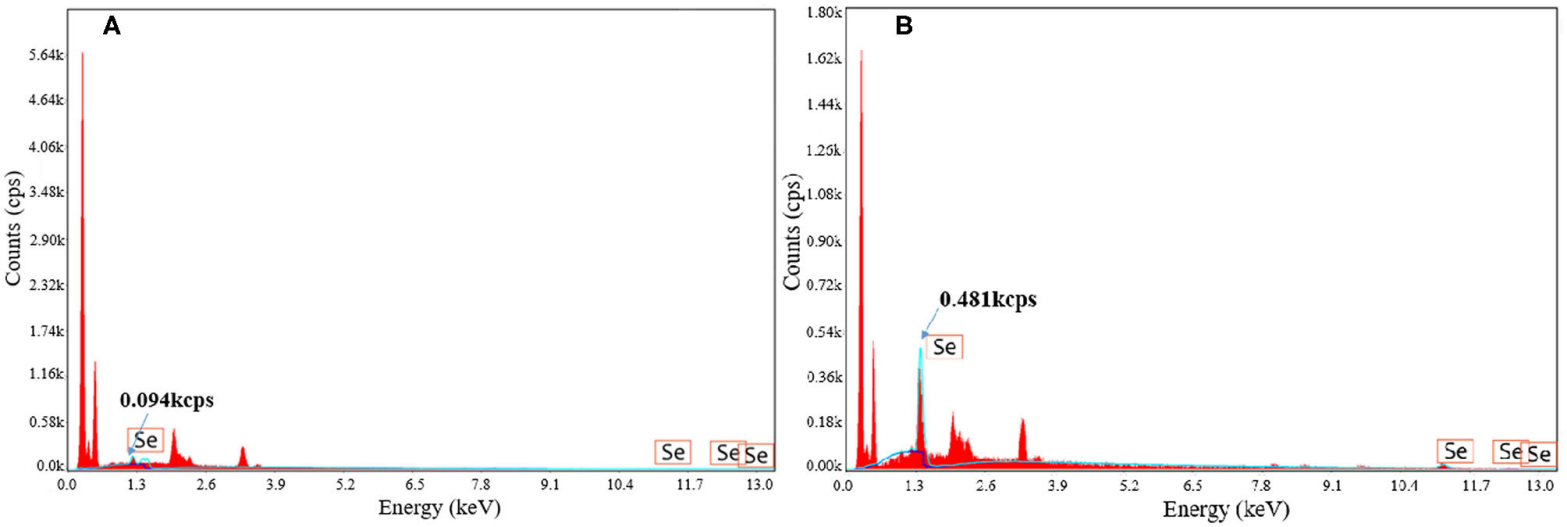
Element analysis with EDS with **(A)** and without **(B)** selenium enrichment.

The surface element analysis of strains with and without selenium enrichment via fermentation was analyzed by energy dispersive spectrometry (EDS) (shown in Figure 3). Compared to strains without selenium enrichment, strains after selenium enrichment showed obvious selenium element peak. The intensity of selenium peak at 1.3 keV increased from 0.094 to 0.481 kcps. Energy spectrum analysis provides evidence that the accumulated selenium became the surface material of the strain after fermentation, showing the selenium enrichment ability of *B. cereus*. Yang et al. (22) also found that there were selenium particles on the surface of the selenium-enriched lactic acid bacteria by scanning electron microscopy-energy spectrum analysis. Xu et al. (23) obtained similar results by scanning electron microscopy and energy spectrum analysis of selenium-enriched lactic acid bacteria. Ying et al. (24) observed the selenium-enriched *Lactobacillus rhamnosus* by transmission electron microscopy and found that there were small black spherical particles with the size of 20–60 mm on the surface and around the bacteria, which was consistent with the size of elemental selenium, and it was identified as elemental selenium by energy dispersive spectrometry. The same phenomenon was also found in *Rhodospirium rubrum, Rhodobacterium sphaeroides, Ralstonia metallifera*, and *Stenotrophomonas maltophilia* (25–28). However, the mechanism needs to be further studied.

## Conclusions

In this study, *B. cereus* was cultured for selenium enrichment. The response surface methodology (RSM) was applied to obtain the maximal selenium conversion ratio (94.3 ± 0.2%), under the optimal fermentation parameters: inoculation quantity 7%, culture temperature 33°C, and shaking speed 170 rpm. The energy dispersive spectrometry analysis showed that inorganic selenium in the form of sodium selenite was transformed into organic selenium, as well as Se^0^ on the surface material of *B. cereus*. This study may contribute to obtain a *B. cereus* strain with high Se conversion ratio, so as to extract organic selenium in the form of selenoprotein for further work. However, due to the fact that *B. cereus* could generate imbalance in human gut since it can produce bacteriocin that inhibits the growth of other beneficial strains, the selenium-enriched *B. cereus* obtained in this study cannot be used directly as human nutrition supplement like the selenium-enriched yeast and *Lactobacillus*, leading to further investigation for valorization of organic selenium in *B. cereus*.

## Data Availability Statement

The raw data supporting the conclusions of this article will be made available by the authors, without undue reservation.

## Author Contributions

XCo, TY, and ZZ: conceptualization. XCh and SL: methodology. XCh: investigation, data curation, and writing—original draft preparation. ZZ: resources. XCh, ZZ, FB, KM, and CP: writing—review and editing. SL and ZZ: supervision. XCo, TY, and SC: project administration. SC: funding acquisition. All authors: have read and agreed to the published version of the manuscript.

## Conflict of Interest

XCo and TY were employed by the company Enshi Se-Run Health Tech Development Co., Ltd. The remaining authors declare that the research was conducted in the absence of any commercial or financial relationships that could be construed as a potential conflict of interest.
